# The Use of Hides during and after Calving in New Zealand Dairy Cows

**DOI:** 10.3390/ani10122255

**Published:** 2020-11-30

**Authors:** Gosia Zobel, Kathryn Proudfoot, Vanessa Cave, Frances Huddart, James Webster

**Affiliations:** 1 AgResearch Ltd., Ruakura Research Centre, 10 Bisley Road, Private Bag 3123, Hamilton 3214, New Zealand; Vanessa.Cave@agresearch.co.nz (V.C.); frankie.huddart@agresearch.co.nz (F.H.); jim.webster@agresearch.co.nz (J.W.); 2 Animal Welfare Centre at the Atlantic Veterinary College, The University of Prince Edward Island, 550 University Ave, Charlottetown, PE C1A 4P3, Canada; kproudfoot@upei.ca

**Keywords:** welfare, maternal behavior, parturition, natural behavior, isolation

## Abstract

**Simple Summary:**

Dairy cows are known to seek isolation during calving. While some regions in the world provide individual calving pens, in pasture-based systems, cows frequently calve in large outdoor groups. We aimed to determine how provision of a “hide” would impact choice of calving location and behavior of the cow and calf. The hides in this study were designed with farmer-utility in mind and were built of heavy-duty gates that could fold out of the way when not needed. When opportunity for seclusion was available, approximately 20% of the cows calved within a hide; however, post-calving seclusion was frequently sought, with over half of the cow–calf pairs moving into hides. When cows and calves isolated after calving, their interaction with other animals was reduced. Hide presence resulted in more dispersed calving location compared to when hides were not available. Factors such as increasing stocking density, and cow age, reduced hide use. This work demonstrates that group housed cows should be provided with a choice regarding calving location.

**Abstract:**

Isolation during calving is a common dairy cow behavior, however it has not been examined in large outdoor group settings. The provision of “hides” was monitored for its impact on calving location and cow–calf behavior. Stocking density and bedding management were either controlled (Phase 1) or managed according to farm practice (Phase 2). Hides were used for calving by 18% (Phase 1) and 22% (Phase 2) of the cows; a further 59% and 44% of cows moved into the hides after calving (Phase 1 and 2, respectively). When hides were not available, cows calved near the edges of the calving area. In Phase 2, as stocking density increased, cows tended to use the hides less. Older cows were less likely to isolate regardless of management. Cow–calf interaction with other cows and calves was lower when hides were available. There was no evidence that hides reduced incorrect matching of cows and calves by staff, however cases of “mismothering” (i.e., calves being taken by other dams) were observed. Since the majority of cows used the hides at some stage before or after calving, we suggest opportunities for seclusion should be provided in large calving groups.

## 1. Introduction

As herd animals, cows synchronize their behaviors and seldom stray far from the protection of the group [1]; however, in the hours before giving birth, cows isolate themselves from herd mates to find a secluded area for parturition. This behavior has been noted in both indoor and outdoor kept cows [2,3,4], and is common in wild ungulates as well. It has been suggested to originate as a safety measure (e.g., to hide the calf from predators), as well as a way for the dam and calf to form a bond without disruption from other cows [5,6]. In many parts of the world, dairy cattle are housed in artificial environments, limiting or preventing the expression of many natural behaviors, including choice in birthing location. Indeed, most cows in North America and Europe are kept in individual or small group pens around parturition [7,8]. Comparably, pasture-based systems (e.g., New Zealand, Australia, Ireland) provide more opportunities for expression of natural behaviors (e.g., grazing; [9]), yet they still require that management decisions be actively made in order to facilitate cows’ desire for seclusion. In New Zealand, for instance, the Code of Welfare’s minimum standards for calving indicate that cows must be allowed to separate themselves [10], however, more information is required regarding how to achieve this in the various options available for calving in pasture-based systems.

When cows calve in paddocks or “stand-off” pads, human disturbance is minimized when the cow is in labor; nonetheless, there are challenges associated with calving in large groups. For example, Illmann and Spinka [11] found that approximately 30% of calves were nursed by a cow other than their dam (i.e., “mis-mothered”) when they were born in a group maternity pen compared with an individual pen. In systems which rely, at least in part, on the dam as a source of colostrum delivery, mis-mothering may have negative health implications for the calf. We suggest that this outcome could be prevented if we better understand the factors that drive cows to calve away from others and to isolate their calf.

To date, research on maternal isolation at calving in dairy cows has focused on indoor maternity areas that allow for one or two cows to be housed together [3,4] or in small groups [12]. For example, Proudfoot et al. [3] designed a maternity pen that allowed cows to calve in an “open” sawdust-bedded pack within sight of other cows, or in a “shelter” surrounded by walls on all four sides except for a door which allowed free access in or out. Most cows used the shelter to give birth, especially during the daytime. When a second cow was added to the pen, this preference reversed; cows were more likely to calve in the open area when they had a partner in the pen. These findings suggest that in enclosed spaces, cows seek seclusion to give birth, but this behavior is impacted by the presence of another cow. Therefore, it is possible that a similar behavioral need exists in dairy cows calving in larger groups, and systems which do not allow for it may impact welfare.

It is unclear if cows housed in large outdoor group pens seek isolation from the herd when given the opportunity, and if the presence of hides could reduce mis-mothering behavior by providing more seclusion to the cow–calf pair. Thus, the objectives of this study were to: (1) determine cow preference for a calving site in a large outdoor group pen with and without the provision of hiding spaces, (2) describe behavioral interactions between calves, their dams, and other cows in these two environments, and (3) assess cow preference for calving site in the same group environment when managed according to standard farm procedure (e.g., variable stocking density, no bedding management). We hypothesized that cows would prefer to use the hides, but that factors such as time of day, and stocking density would affect this preference. We also hypothesized that hides would allow cows and calves to spend more time together and less time with other animals in the group.

## 2. Materials and Methods

This study was undertaken on the AgResearch Tokanui Dairy Research Farm near Hamilton (175°18 00′ E longitude, 38°03 00′ S latitude), New Zealand between August and September 2016 (Southern hemisphere winter). All procedures involving animals were approved by the Ruakura Animal Ethics Committee (AE # 13926) under the New Zealand Animal Welfare Act 1999.

### 2.1. Animals and Facilities

All cows received a unique paint identification mark (Tell Tale animal marker, FIL, GEA Farm Technologies, Mt Maunganui, New Zealand) at enrolment. Cows were managed as per normal Tokanui Research Farm practice for cows that are close to calving. They were kept on pasture between approximately 07:30 and 15:30, then, between approximately 16:00 and 08:00 the following morning, the cows were kept on two uncovered pads (50 m × 20 m), adjacent to each other and surrounded by 3 m solid timber walls. Immediately before, and immediately after, being on pasture the cows were fed a maize silage ration of 12 kg DM/d/cow split between the two feeding periods, which were approximately 30 min in duration.

**Figure 1:**
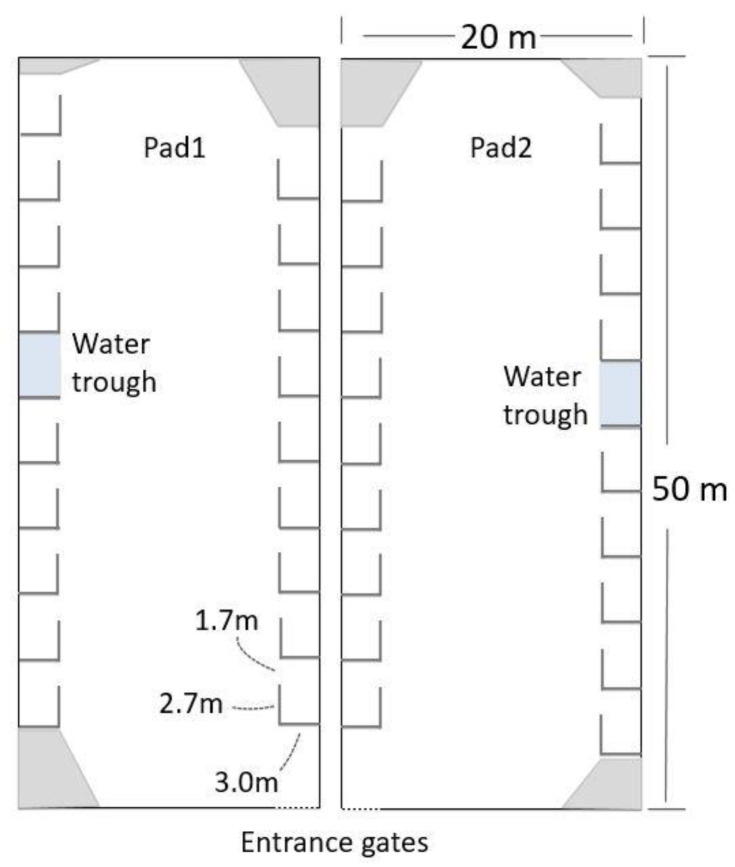
Pads were next to each other. The hides were opened and closed on each pad on alternating days (Phase 1, 22 d) and left open on both pads (Phase 2, 18 d). Grey indicates corners that were walled off with plywood to discourage crowding in corners that were not part of the hides.

Nine hides (Figure 1) were constructed along each long side of the pad, 18 per pad.

**Figure 2:**
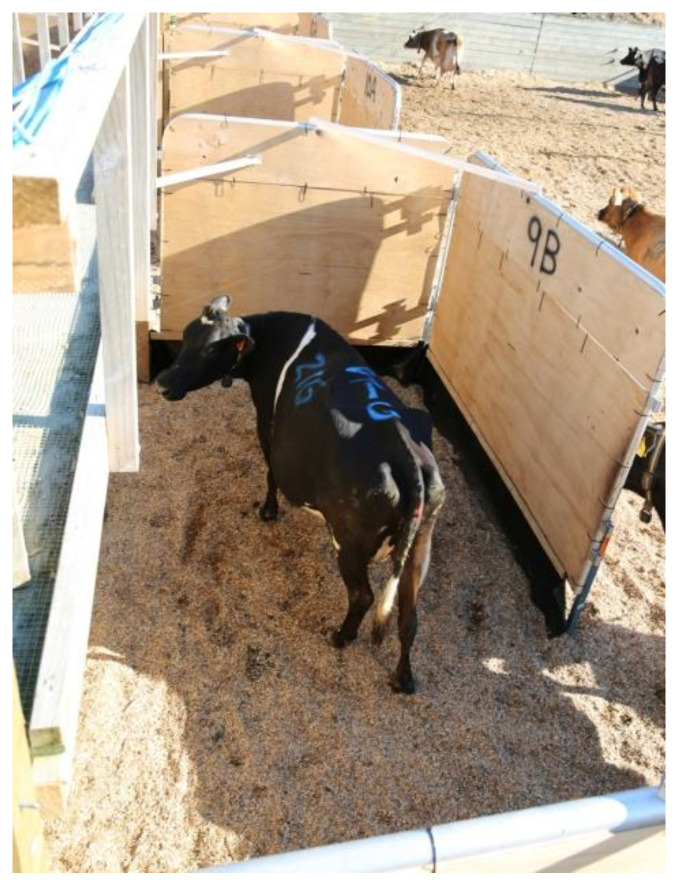
Birds’ eye view of hides constructed from two interlinked gates and covered in plywood and rubber sheeting. Spring-loaded metal foot supported the outer gate, and two aluminum crossbars inserted via a dowel kept the gates open.

A portable interlocking yard system comprised of two gates (2.7 m and 3.0 m, Prattley gates, Prattley Industries Ltd., Temuka, New Zealand) formed the L-shaped hides (Figure 2). Gate gudgeons were fastened to the reinforced timber wall and one gate hung from these such that the bottom was approximately 0.35 m from the ground; the other gate was attached to the first using the linkages supplied with the gates, thus the angle of the gates could be adjusted as required. When the hides were in the open position the gates were at 90° to each other, and a spring-loaded metal foot supported the outer gate. The gates were secured in position by two aluminum crossbars inserted via a metal dowel.

The opening of each hide was 1.7 m wide, allowing easy movement in and out of the hide area. All gates were covered with 9 mm plywood, and 3 mm rubber sheeting (Para Rubber, Hamilton, New Zealand) was suspended from the bottom of the gate to the ground, thus giving visual isolation for the cow and preventing the calf from rolling under the gates. When the hides needed to be closed, the two crossbars were removed, and the gates were folded on top of each other and laid along the wall and held in position by ties to the top of the wall. Plywood was fastened across the corners of each pad to remove the opportunity for the cows to calve in these areas. Each pad had a water trough placed midway along the outer wall. Pads were bedded at a depth of 30 to 40 cm of *pinus radiata* woodchipper fines, passed through a 10 mm × 10 mm steel mesh screen (Mooreys Contracting Ltd., Matamata, New Zealand).

There were two phases to this study. During Phase 1, feces and placentae were removed from the pads daily; at the midway point of this phase, the top 15 cm of bedding was removed from the pads and replaced with fresh bedding. During Phase 2, the pad was managed as per standard Tokanui Research Farm practice, with no cleaning of feces and placentae or bedding refreshing, for the duration of the phase.

#### Calf Handling

For both phases, farm personnel arrived at approximately 05:30 to identify any cows that had calved. To minimize disturbance and the potential of incorrectly assigning calves to the wrong dams, personnel used headlamps instead of turning on the overhead lighting system; however, we acknowledge that worker presence may have affected cow and calf behavior between 05:30 and 08:00. Calves were ear tagged and their dam ID was recorded. A tissue ear punch from the calf was also collected. Calves stayed with their dams until cows were removed for feeding at approximately 08:00. Calves were then removed and cared for according to standard Tokanui Research Farm practice.

### 2.2. Experimental Design

A power analysis performed prior to the study beginning was conducted using Proudfoot et al. [3]. Assuming a desired power of 80% and significance of 5%, it was determined that at least 20 cows per group (e.g., with hides and without) would be needed for examining location preference. For binomial comparisons (e.g., mis-mothering when cows used a hide versus not, only when hides were available), it was determined that at least 44 cows per group would be needed. With the latter in mind, we aimed to enroll as many cows as were available during the study period.

#### 2.2.1. Phase 1

This period was 22-d long. On the first day, 100 Friesian-cross dairy cows were selected from the farm’s larger pre-partum group based on having an imminent calving date. Cows were brought in from pasture in the afternoon and received their standard supplemental silage on the feed pad. After 30 min, the cows were brought to the entrance of the overnight housing pads in small groups, where they were manually drafted alternately one to each pad, randomizing cow assignment. To achieve a stocking density of 20 m^2^/cow, there were always 50 cows assigned per pad. When a cow calved, she was weighed (Gallagher TSI smart scales, Gallagher, Hamilton, New Zealand) and removed from the enrolment group to join the milking herd (as per regular farm protocol). Following this, a new cow which was close to calving was enrolled; therefore, there were always 100 cows on any given day which could calve. The hides within each pad were opened and closed on alternate nights such that when on one pad the hides were open, on the other, they were closed. Over the 22-d period, a total of 298 cows were enrolled, and 198 of these calved. Cows which calved during the day at pasture (*n* = 88), which had stillborn calves (*n* = 2) or which did not have a confirmed calving location due to video malfunction (*n* = 3) were not included in the final dataset; the final dataset contained 105 cows. The median age of these cows was 5 years (min = 2, Q1 = 4, Q3 = 7, max = 13) and their median weight was 485 kg (min = 344, Q1 = 431, Q3 = 524, max = 670).

#### 2.2.2. Phase 2

This 18-d period followed directly after Phase 1 and used a further 71 cows. All cows had a unique paint identification from Phase 1 but had not yet calved. When the cows were brought to the pads, they were counted in by farm personnel, resulting in a dynamic number of cows each night (38 ± 12.1 cows/pad) and a mean stocking density of 29 ± 9.5 m^2^/cow. Daytime management was similar to Phase 1, with the exception that the feed ration was only fed once a day and was present on the pads when the cows arrived in the afternoon. The hides in both pads were always accessible to the cows. For Phase 2, cows were not weighed after calving, as this was outside typical farm management. After excluding cows that calved during the day on pasture (*n* = 30), the final dataset contained 41 cows with a median age of 5 years (min = 2, Q1 = 3, Q3 = 8, max = 11).

### 2.3. Behavioral Observations

Behavior was recorded continuously in real time (30 fps) by a security NVR system (ND9541, Vivotek, Taiwan) and color 4-megapixel, 2.8 mm cameras (DS-2CD2432-F-IW, Hikvision, Hangzhou, China), with built-in infrared light to allow for nighttime video collection in black and white. The cameras were mounted on 100 mm × 100 mm × 6 m wooden poles placed such that in most cases 1 camera focused on 2 hides; the remaining cameras were focused on the rest of the area available to the cows, including a general overview from the back and front of each pad.

For both phases, calving records were reviewed each morning, and the overview cameras were reviewed to locate cows and their approximate calving time. A single observer then used the more focused cameras to determine exact calving time and location. If hides were available, calves were recorded as calving either inside or outside of a hide. To determine hide usage after calving, the cow–calf pairs were followed until either they moved into a hide, or farm personnel arrived to move cows to pasture in the morning. For Phase 1 only, 10 cow–calf pairs that moved into a hide were matched with 10 cow–calf pairs that did not have hide access on the same night. Matches were made based on as similar as possible calving time with a median time between calvings of 44 min (min = 1, Q1 = 22, Q3 = 179, max = 293).

Detailed behaviors (Table 1) were recorded for 30 min prior to the cow–calf pairs moving into the hide, and then 30 min following moving into the hide, or similar time period relative to calving for the matched pair that did not have hide access. Intra-observer reliability was performed on 2 cows, once prior to observation beginning and again at the culmination of all observations. A weighted kappa was calculated (all behaviors combined, before: κw = 0.81; after: κw = 0.84).

### 2.4. DNA Analysis

Calf ear punch tissue samples were frozen within an hour of collection and stored at −20 °C until the end of Phase 1. Four samples were deemed to be insufficient in quality and could not be tested. All remaining samples (*n* = 101) were submitted at once for DNA analysis using GeneMark^®^ methodology [13]. Parentage was confirmed using the existing dam database, managed by NZ’s Livestock Improvement Corporation.

### 2.5. Data Handling and Statistical Analysis

All statistical analyses were performed in Genstat 19th Edition. For each Phase, the percentage of cows that chose to calve in a hide was calculated, with 95% Binomial confidence intervals. Logistic regression was used to assess whether there was evidence that probability a cow used a hide to calve was affected by the pad, the time of calving, the cow’s age, the number of days of exposure to the pad before calving, whether those previous exposure days had hides open or the post-calving body weight of the cow. The statistical significance of each variable was assessed using backwards elimination and the change in deviance. Similarly, the percentage of cow–calf pairs that moved into a hide after calving was calculated, and the statistical significance of each variable was assessed. For Phase 1 only, to provide a descriptive representation of calving distribution when hides were available or when they were not available, daily calving locations on each pad were plotted. These locations were also animated to illustrate the cow–calf pairs that moved after calving. In addition, a maximum likelihood chi-square contingency table test used to test for an association between hide access and calving location (classified as the front, middle or back of the pad). Due to sparsity of data, it was pooled across both pads. Furthermore, for Phase 1 only, the effect of hide access on behavior of cow–calf pairs during the 30-min periods before and after moving into the hides was assessed using a 2-way ANOVA blocked by the matched pair and the cow–calf pair within the matched pair. Finally, for Phase 1 only, a two-sided, two-sample Binomial test was used to examine whether access to hides impacts the percentage of calves successfully assigned dams.

## 3. Results

### 3.1. Phase 1

#### 3.1.1. Calving Location

**Figure 3:**
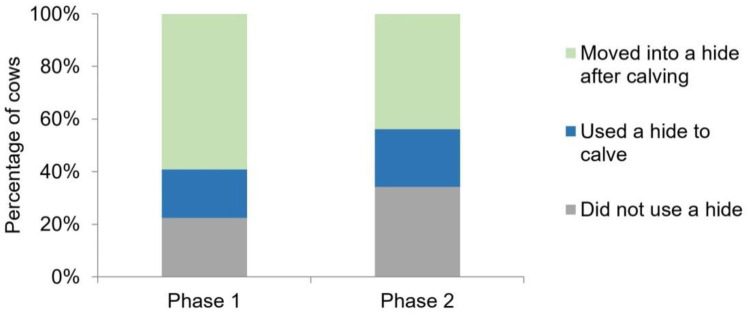
The percentage of cows and cow–calf pairs that did not use a hide, cows that calved in a hide, and cow–calf pairs that moved into a hide after calving during two phases of the study. Phase 1 (*n* = 49) had consistently managed stocking density, daily bedding cleaning, and random allocation of cows to 1 of 2 calving pads each night. Phase 2 (*n* = 41) followed the farm’s standard practice, with variable stocking density on both calving pads, and no bedding maintenance.

Approximately half of the cows calved when the hides were available (49/105; 47%). Out of those 49 cows, 9 chose to calve in a hide (18%, CI: 8–29; *p* < 0.001; Figure 3). There was no evidence that hide use for calving was affected by the pad (*p* = 0.28), the time of calving (*p* = 0.79), the cow’s age (*p* = 0.92), the number of days of exposure to the pad before calving (*p* = 0.95), whether those previous exposure days had hides open (*p* = 0.18), or the post-calving body weight of the cow (*p* = 0.67).

#### 3.1.2. Calving Distribution

**Figure 4:**
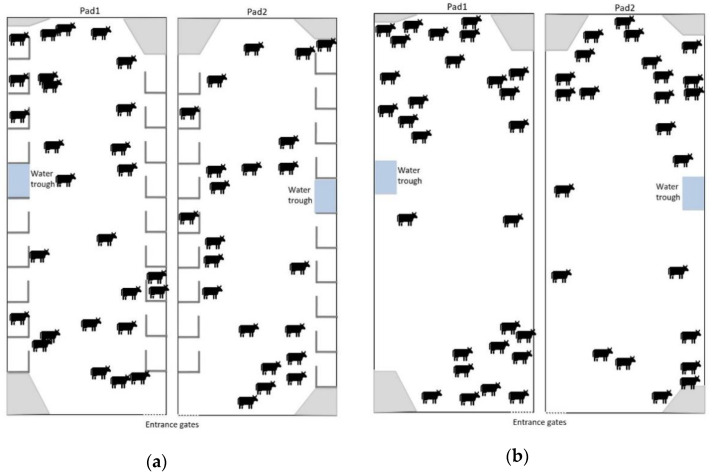
Plots of calving location during Phase 1: (**a**) When hides were open (*n* = 49); (**b**) When hides were closed (*n* = 52). Stocking density was standardized at 50 cows/pad, bedding was cleaned daily and cows were randomly allocated to calving pads each night. Hides were opened and closed on alternating nights on each pad. Cows were on pasture together during the day.

Whether a cow had access to a hide impacted whether she calved in the front, middle or back of the pad (χ^2^ = 6.21; *p* = 0.04); cows that calved with hides available were more likely to calve in the middle of the pad compared to those without access to hides (27% vs. 11%, respectively), and those without access to a hide were more likely to calve in the back of the pad compared to those with access to a hide (52% vs. 33%, respectively). Calving locations are presented descriptively for when hides were available (Figure 4a) and when hides were not available (Figure 4b) on both pads.

#### 3.1.3. Moving after Calving

**Figure 5:**
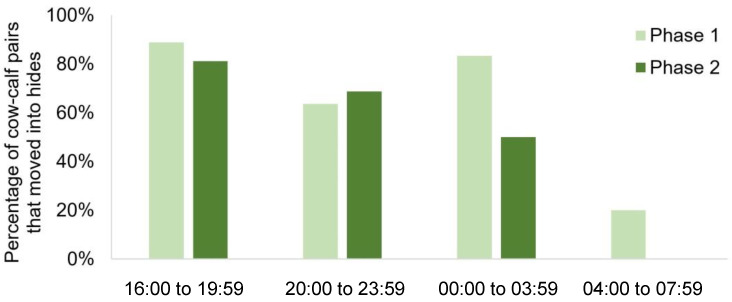
The percentage of cow–calf pairs that moved into hides after calving in the late afternoon, evening, middle of the night or early morning for both phases of the study. Phase 1 (*n* = 49) had consistently managed stocking density, daily bedding cleaning, and random allocation of cows to 1 of 2 calving pads each night. Phase 2 (*n* = 41) followed the farm’s standard practice, with variable stocking density on both calving pads, and no bedding maintenance. Note: Results for 04:00 to 07:59 could have been impeded by the presence of farm personnel at approximately 05:30.

Of the 40 cows that did not calve in hides, 73% (29/40) of the cow–calf pairs moved into a hide after calving (CI: 59–86%; *p* = 0.006; Figure 3). The probability of a cow–calf pair moving into a hide was affected by the time of day of calving (*p* = 0.002; Figure 5); cow–calf pairs were most likely to move into a hide if calving in the early evening to the middle of the night (16:00 to 19:59: 89% of pairs moved; 20:00 to 23:59: 64% of pairs moved; 00:00 to 03:59: 83% of pairs moved) and were the least likely to enter a hide if calving in the early morning (04:00 to 08:00; 20% of pairs moved); however, we caution that farm personnel presence at 05:30 could have impacted the latter result. Median latency for cow–calf pairs to move into a hide was 2.4 h (min = 1.0, Q1 = 1.7, Q3 = 3.5, max = 8.7). Older cows moved into hides less; every 1 year increase in age tended to decrease the log odds of moving into a hide after calving by 0.52 ± 0.36 (*p* = 0.09). Pad also tended to affect cow–calf pair movement (Video S1); the log odds of moving into Pad 2 were 2.2 ± 1.3 higher than Pad 1; *p* = 0.053).

#### 3.1.4. Cow–Calf Behavior

There was no evidence of effects of hide access on any of the behavior of cow–calf pairs during the 30-min periods before and after moving into the hides (Table 2). Regardless of hide access, there was a tendency for cow–calf pairs to have longer bouts interacting with other cows or calves during the 30-min period before moving into a hide (0.19 vs. 0.12 ± 0.04%; *p* = 0.096). There was an interaction between hide access and period for the bout duration of movements initiated by the dam or calf, as well as on the percentage of time cow–calf pairs interacted with other cows or calves in the pen (Table 2).

Cow–calf pairs with access to a hide had longer movement bouts during the 30-min period after moving into the hide compared to before, whereas cow–calf pairs with no hide access showed the opposite response (*p* = 0.02). Furthermore, when cow–calf pairs had hide access, they spent less time interacting with other cows or calves in the 30-min period after they moved into the hide, compared to before, whereas cow–calf pairs without hide access showed the opposite response (*p* = 0.02). Cow–calf pairs with hide access also tended to have fewer (*p* = 0.09) and shorter (*p* = 0.07) interactions with other cows or calves after moving into the hide.

#### 3.1.5. Cow and Calf Matching

Overall, 78% (79/101) of calves had successfully assigned dams (CI: 69–86%; *p* < 0.001). Of these, 32% (7/22) were observed from video recordings to be actively “stolen” by another dam. There was no evidence that having access to hides impacted the number of calves that were allocated by staff to the wrong dams (*p* > 0.1); 22% of the calves born to dams with hide access were incorrectly matched, and 20% were incorrectly matched when no hides were available.

### 3.2. Phase 2

#### 3.2.1. Calving Location

When cows were managed under normal farm practice (e.g., no fresh bedding, dynamic stocking density), and the hides were always available while the cows were on the pads, 22% (9/41) of the cows calved in the hides (CI: 9–35; *p* < 0.001; Figure 3). Age and stocking density affected the probability that a cow calved in a hide. For every 1 year increase in age, the log odds of calving in a hide decreased by 0.54 ± 0.24 (*p* = 0.006), and for each increase in stocking density (i.e., one extra cow per m^2^) decreased the log odds of calving in a hide by 0.13 ± 0.06 (*p* = 0.02).

#### 3.2.2. Moving after Calving

Of the 32 cows that did not calve in a hide, 18 cow–calf pairs moved into hides after calving (56%; *p* = 0.60; Figure 3). Similarly to Phase 1, the time of day (*p* = 0.04; Figure 5) impacted whether cows moved into hides after calving; cow–calf pairs were most likely to move into a hide in the early to late evening (16:00 to 19:59: 75% of pairs moved; 20:00 to 23:59: 62% of pairs moved) and were the least likely to move into a hide in the early morning (00:00 to 03:59: 50% of pairs moved; 04:00 to 08:00: 0% of pairs moved); as with Phase 1, the presence of farm personnel at 05:30 could have impacted this result. Median latency for cow–calf pairs to move into a hide was 2.7 h (min = 0.4, Q1 = 1.8, Q3 = 4.0, max = 7.0). The probability that a cow–calf pair moved into a hide after calving also tended to be negatively influenced by increasing stocking density of the pen (*p* = 0.054).

## 4. Discussion

We evaluated whether cows in large outdoor groups would seek out isolation for calving. We also aimed to establish how availability of hiding opportunity would impact cow–calf interactions. In both of our study phases (i.e., consistent stocking density paired with routine cleaning of bedding versus dynamic stocking density and no bedding management), most cows did not calve in the hides. These findings are contrary to other studies focusing on indoor housed cows. Two studies using indoor individual maternity pens found that Holstein dairy cows were more likely to use a hiding space during labor [3,4]. The discrepancy may be partially due to the difference in group composition of the calving pens (individual vs. large dynamic groups), or the time of day that cows calved. For example, cows calving in pairs or overnight were less likely to use a hide than cows which were housed alone and calved during the day [3]. In our study, cows calved in a large group, mainly during the evening and night when the motivation to hide may be less than during the day. A lower motivation to hide overnight has been seen in wild elk; Dzialak et al. [14] found that when elk living near human activity (i.e., a gas field) calved during the daytime, their calving sites were characterized by natural cover and avoidance of the gas field, however, if elk calved at night they showed no preference for cover or distance from the human activity.

Within the cows that were managed according to standard farm practice (Phase 2), older cows were less likely to calve in a hide than younger cows. It is unclear why age affected hide use, but may be driven by social hierarchy within the group or previous experience. For instance, in a small group setting, Rorvang et al. [15] found that approximately half of the cows chose to calve in a hide, and that these cows were more likely to be the dominant individuals at the feedbunk. Age contributes substantially to social hierarchy in cattle [16], indirectly suggesting that hierarchy could have played a role in our study; interestingly, we found the opposite to Rorvang et al. [15]. Previous experience could explain these findings. For example, in a study using group-housed cows on pasture with access to a barn, Edwards et al. [12] found that younger, primiparous animals were more likely to segregate from herdmates and calve in an area of natural seclusion (e.g., tall grass and treecover), whereas older animals were more likely to calve in the barn. The authors speculate that older cows may have calved in a barn previously, which may have impacted their choice of calving site. Our cows were entirely pasture-based; the only enclosed space they would have experienced (after a few weeks of age) would have been the milking parlor; this may or may not be a pleasant environment for them, thus they may not see value in enclosed areas. Furthermore, many of the cows in the current study had calved numerous times without access to seclusion (e.g., the median age was 5 years, and ranged from 2 to 13 years); it is therefore possible that hiding instincts in older cows had been suppressed by the lack of previous seclusion opportunities. Ideally, future research would enroll heifers and follow these animals over multiple calvings, in order to address the potential of familiarity and previous experience dictating calving location decisions.

Cows were more likely to position themselves against a wall compared to the open part of the pen, as evidenced when the hides were not available. Thus, cows may have wanted some seclusion while giving birth, however, the particular design of our hides could have been too isolating for cows to remain vigilant during labor. By using dimensions described in North American and European Holstein research, our hides provided ample space for the New Zealand crossbred dairy cows used in the current study; however, cows may have sought more visual connection to the rest of the herd regardless of space allowance. In general, cows are averse to complete social isolation from the protection of the herd, as they show physiological and behavior signs of stress when they are completely isolated from herd mates [17]. In a natural setting, cows isolate from the herd at calving, but they will do so in tall grasses or tree cover [2]; this likely provides them with the ability to see herd mates during labor. The fact that at least some cows did use hides suggests that further hide designs should be explored in future work; we recommend a gate style set-up like was used in the current study, as these could be folded away when not in use (Video S2); however, we suggest that perhaps the upper panels could be left open, to allow the cow to maintain some visual contact with her surroundings. Alternatively, single-sided barriers positioned against the wall, or throughout the pen, may encourage more hiding behavior, such as those described by Creutzinger et al. [18].

A novel finding from this study was that a majority of cow–calf pairs moved into hides after calving. It is unclear whether this behavior is driven by the cow or the calf, but regardless of who was motivated to move, it was likely due to a need to hide the calf. Like many other ungulate species, cows will hide their calves after parturition [2]. In modern production systems, human caretakers will find and remove the calf from their dam in early life. However, cattle are thought to be either “hider” or “follower” species, where the calf will either remain hidden from the herd for a short period of time after birth, or will follow the dam either immediately or soon after birth (see review [6]). For example, in feral Maremma cattle, calves were observed hiding in brush for the first 2 to 3 days of life while their dams grazed nearby; the calves would then follow their dams for the next few days, and finally would join the herd after about a week of life [19]. The calves in the present study would need to be observed longer (e.g., all calves were separated from their dams the morning following their birth), in order to determine how long they sought to stay secluded.

Older cows and their calves tended to be less likely to use a hide after calving. There is no evidence of age affecting maternal behavior in beef cattle [20] or dairy cattle [21]. Older beef dams were found to spend less licking and following calves compared to younger dams, but spend more time nursing [20]. Similarly, younger dairy cows had a shorter latency to sniff their calves, and spent more time sniffing and licking compared to cows of later parities [21]. Beef cows did show individual differences in maternal behavior, but these differences are driven more by calf sex (male calves were given more care than female calves), calf birth weight (lighter calves were given more care), and cow body condition (the better condition, the more care; [20]). Therefore, regardless if it is the calf or the cow driving the hiding behaviour, we suggest that calves born to older cows may require less intensive care from the cow, and thus the pair are less likely to seek out shelter. Furthermore, cow–calf pairs were more likely to move into a hide if they calved late at night compared to early in the morning. While there were fewer early morning calvings in general (e.g., six cows in Phase 1, and 3 cows in Phase 2 calving after 04:00), this finding may also be due to late night calvers having more time than morning calvers to move. On average, it took cow–calf pairs 2 h to move into a hide; therefore, movement of cows having calved early in the morning was likely to have been disrupted by farm staff arrival. Cow–calf pairs may also have been more motivated to move into a hide in the late evening when diurnal activity is highest [22] and the risk of predation may be greater compared to later hours.

Moving into hides after calving decreased the interactions that cow–calf pairs had with other cows and calves. Cows, particularly those that are close to calving, are attracted to pheromones [23] and amniotic fluid of other recently calved cows [24,25]. Reducing outside interference from other cows is important; it allows the dam to direct her attention to her calf during the first few hours after birth [21]. When other cows are present, licking is reduced [23], and nursing is disrupted (e.g., 30% of calves were nursed by other dams; [11]). The latter has the potential to be extremely detrimental to the health of the calf, as colostrum quality is best immediately following calving [26], and successful passive transfer of immunity is highest if the calf consumes good quality colostrum within the first 2 h after birth [27]. In New Zealand, cows are often the first source of colostrum, even if additional colostrum is provided after separation (e.g., in a survey of 101 farms, 65% reported picking up calves once a day or less; [28]). In Phase 1 of our study, where parentage could be determined, 22 calves were not assigned to the correct dam by staff; during video scanning, seven of these calves were observed to be actively “mis-mothered”, where another cow pushed the dam away and spent the rest of the night with the calf. Unfortunately, this frequency of mis-mothering likely impeded the power to evaluate whether hide presence could have reduced this behaviour. We suggest follow-up work with more cows to explore not only benefits of provision of seclusion opportunities, but also its impacts on serum IgG of the calves (i.e., to determine the extent to which such behaviours impact calf health).

When cows were managed according to standard farm practice in Phase 2, hides were used less with increasing stocking density. While this was contrary to our hypothesis, we propose a few explanations. First, although not quantified, the cows did use the hides for behaviours other than calving; while scanning the video for calving location, we observed non-calving cows resting, scratching and spending idle time in the hides. Without daily cleaning of the bedding, these areas may have become undesirable calving locations, as cows are known to avoid wet and dirty bedding when given the option [29]. Second, cows could choose their social group during this phase. Even though all cows were pastured together during the day in both phases, it is nonetheless possible that during the first phase, where cows were randomly allocated to each pad, we were creating a disrupted social grouping each night, therefore promoting cows to seek out isolation. When cows were staying in stable social groups, they may have been feeling more secure and less motivated to seclude themselves.

## 5. Conclusions

Just over 20% of the cows chose to calve in the provided seclusion opportunities, yet the majority of those that did not use them initially, moved there with their calves in the hours following calving. Depending on management (either cleaned or non-cleaned bedding, and consistent or dynamic stocking density), various factors impacted hide use, including stocking density, age and time of day. Further work would be needed to tease these apart, as well as to better understand best hide design and how hide use impacts calf health. This aside, it is important to recognize that promoting good animal welfare includes providing animals with choice. Over two-thirds of the cows in this study chose to use the provided hides at some point during or after calving, therefore, we suggest that pre- and post-partum cows should be given the opportunity to isolate.

## Figures and Tables

**Table 1:** Ethogram for coding interactions between cow–calf pairs and other cows and calves. Ten pairs which moved into hides where matched with 10 pairs that did not have hide access, but that calved within a similar timeframe. Behaviors were coded 30 min before moving into the hide, and 30 min after moving into the hide. Before and after periods for cow–calf pairs without access to a hide were based on their matched pairs’ hide entrance time relative to calving.

Behavior	Definition
Movement initiated by cow or calf	Either the cow or the calf initiates movement, 3 legs must be moving, must be upright (excludes repositioning while lying).
Interactions between cow–calf pair	Calf and cow are standing or lying together within one cow length of one another; cow or calf is either licking, rubbing or pushing with nose, or the calf is standing facing towards the udder of the cow.
Interaction with other cows or calves	Either the cow or the calf interacts with another cow or calf that is not part of the cow–calf pair, via licking, rubbing or pushing with the nose.

**Table 2:** Behavior between 10 cow–calf pairs were recorded for the 30 min period before, and 30 min period after, moving into a hide; cow–calf pairs without hide access were matched to those that had hide access based on similar calving time.

Behavior	No Hide Access		Hide Access		SED	*p*-Value _(hide)_	*p*-Value _(period)_	*p*-Value _(hide X period)_
	Before ^1^	After ^1^	Before	After				
Movement initiated by cow or calf								
% of 30 min period	11.3	11.1	14.5	11.6	3.6	0.54	0.47	0.52
Bouts (no./30 min)	31.1	33.5	38.9	27.8	10.9	0.91	0.49	0.29
Bout duration (min/bout)	0.12	0.09	0.11	0.14	0.02	0.17	0.85	0.02
Interactions between cow–calf pair								
% of 30 min period	32.2	34.1	47.4	30.7	10.2	0.50	0.22	0.13
Bouts (no./30 min)	22.7	26.4	36.0	24.4	7.3	0.33	0.43	0.14
Bout duration (min/bout)	0.50	0.37	0.41	0.34	0.11	0.53	0.12	0.62
Interaction with other cows/calves								
% of 30 min period	5.5	8.2	4.6	0.9	5.0	0.42	0.65	0.01
Bouts (no./30 min)	7.6	12.2	5.4	1.9	4.5	0.15	0.81	0.09
Bout duration (min/bout)	0.18	0.18	0.20	0.05	0.08	0.47	0.10	0.07

^1^ “before” and “after” periods for cow–calf pairs without access to a hide were based on their matched pairs’ hide entrance time relative to calving.

## Supplementary Materials

The following are available online at https://www.mdpi.com/2076-2615/10/12/2255/s1, Video S1: Calving locations and the hides to which cows moved following calving (Phase 1 only), Video S2: Hide closing demonstration.

## References

[1] Miller K., Wood-Gush D.G.M. (1991). Some effects of housing on the social behaviour of dairy cows. Anim. Sci..

[2] Lidfors L.M., Jensen P., Algers B. (1994). Suckling in Free-ranging Beef Cattle—Temporal Patterning of Suckling Bouts and Effects of Age and Sex. Ethology.

[3] Proudfoot K.L., Weary D.M., Von Keyserlingk M.A.G. (2014). Maternal isolation behavior of Holstein dairy cows kept indoors. J. Anim. Sci..

[4] Proudfoot K.L., Jensen M.B., Weary D.M., Keyserlingk M.A.G. (2014). Von Dairy cows seek isolation at calving and when ill. J. Dairy Sci..

[5] Lott D.F., Galland J.C. (1985). Parturition in American bison: Precocity and systematic variation in cow isolation. Z. Tierpsychol..

[6] Rørvang M.V., Nielsen B.L., Herskin M.S., Jensen M.B. (2018). Prepartum maternal behavior of domesticated cattle: A comparison with managed, feral, and wild ungulates. Front. Vet. Sci..

[7] United States Department of Agriculture (USDA) (2016). Dairy Cattle Management Practices in the United States, 2014.

[8] Fogsgaard K.K., Herskin M.S., Gorden P.J., Timms L.L., Shearer J.K., Millman S.T. (2016). Management and design of hospital pens relative to behavior of the compromised dairy cow: A questionnaire survey of Iowa dairy farms. Appl. Anim. Behav. Sci..

[9] Mee J.F., Boyle L.A. (2020). Assessing whether dairy cow welfare is “better” in pasture-based than in confinement-based management systems. N. Z. Vet. J..

[10] New Zealand National Animal Welfare Advisory Committee (2018). Code of Welfare: Dairy Cattle.

[11] Illmann G., Špinka M. (1993). Maternal behaviour of dairy heifers and sucking of their newborn calves in group housing. Appl. Anim. Behav. Sci..

[12] Edwards E.M., Krawczel P.D., Dann H.M., Schneider L.G., Whitlock B., Proudfoot K.L. (2020). Calving location preference and changes in lying and exploratory behavior of preparturient dairy cattle with access to pasture. J. Dairy Sci..

[13] Livestock Improvement Corporation DNA Parentage Testing—GeneMark. https://www.lic.co.nz/products-and-services/animal-health-and-dna-testing/dna-parentage-testing/.

[14] Dzialak M.R., Harju S.M., Osborn R.G., Wondzell J.J., Hayden-Wing L.D., Winstead J.B., Webb S.L. (2011). Prioritizing conservation of ungulate calving resources in multiple-use landscapes. PLoS ONE.

[15] Rørvang M.V., Herskin M.S., Jensen M.B. (2018). The motivation-based calving facility: Social and cognitive factors influence isolation seeking behaviour of Holstein dairy cows at calving. PLoS ONE.

[16] Šárová R., Špinka M., Stěhulová I., Ceacero F., Šimečková M., Kotrba R. (2013). Pay respect to the elders: Age, more than body mass, determines dominance in female beef cattle. Anim. Behav..

[17] Rushen J., Boissy A., Terlouw E.M.C., De Passillé A.M.B. (1999). Opioid peptides and behavioral and physiological responses of dairy cows to social isolation in unfamiliar surroundings. J. Anim. Sci..

[18] Creutzinger K.C., Dann H.M., Moraes L.E., Krawczel P.D., Proudfoot K.L. (2020). Effects of prepartum stocking density and a blind on physiological biomarkers, health, and hygiene of transition Holstein dairy cows. J. Dairy Sci..

[19] Vitale A.F., Tenucci M., Papini M., Lovari S. (1986). Social behaviour of the calves of semi-wild Maremma cattle, Bos primigenius taurus. Appl. Anim. Behav. Sci..

[20] Stěhulová I., Špinka M., Šárová R., Máchová L., Kněz R., Firla P. (2013). Maternal behaviour in beef cows is individually consistent and sensitive to cow body condition, calf sex and weight. Appl. Anim. Behav. Sci..

[21] Jensen M.B. (2012). Behaviour around the time of calving in dairy cows. Appl. Anim. Behav. Sci..

[22] Albright J.L. (1993). Feeding Behavior of Dairy Cattle. J. Dairy Sci..

[23] Edwards S.A. (1983). The behaviour of dairy cows and their newborn calves in individual or group housing. Appl. Anim. Ethol..

[24] Pinheiro Machado F L.C., Hurnik J.F., Burton J.H. (1997). The effect of amniotic fluid ingestion on the nociception of cows. Physiol. Behav..

[25] Rørvang M.V., Nielsen B.L., Herskin M.S., Jensen M.B. (2017). Short communication: Calving site selection of multiparous, group-housed dairy cows is influenced by site of a previous calving. J. Dairy Sci..

[26] Moore M., Tyler J.W., Chigerwe M., Dawes M.E., Middleton J.R. (2005). Effect of delayed colostrum collection on colostral IgG concentration in dairy cows. J. Am. Vet. Med. Assoc..

[27] Chigerwe M., Tyler J.W., Schultz L.G., Middleton J.R., Steevens B.J., Spain J.N. (2008). Effect of colostrum administration by use of oroesophageal intubation on serum IgG concentrations in Holstein bull calves. Am. J. Vet. Res..

[28] Cuttance E.L., Mason W.A., Laven R.A., Denholm K.S., Yang D. (2018). Calf and colostrum management practices on New Zealand dairy farms and their associations with concentrations of total protein in calf serum. N. Z. Vet. J..

[29] Schütz K.E., Cave V.M., Cox N.R., Huddart F.J., Tucker C.B. (2019). Effects of 3 surface types on dairy cattle behavior, preference, and hygiene. J. Dairy Sci..

